# The Effectiveness of a Parent-Training Program for Promoting Cognitive Performance in Preschool Children

**DOI:** 10.5964/ejop.v13i3.1381

**Published:** 2017-08-31

**Authors:** Elahe Vahidi, Amir Aminyazdi, Hossein Kareshki

**Affiliations:** a Department of Education and Psychology, Ferdowsi University of Mashhad, Mashhad, Iran; Webster University Geneva, Geneva, Switzerland

**Keywords:** parent training program, parent-child interactions, preschool children, cognitive performance

## Abstract

The study aims to evaluate the effectiveness of a parent training program for promoting cognitive performance of young children through enriching the parent-child interactions among mothers of preschool-aged children in Mashhad, Iran. A total of 29 couples of mothers and their children were assigned to an experimental group (n = 16 couples) and a control group (n = 13 couples). Mothers in the experimental group participated in 12 weekly sessions and were trained how to enrich their daily parent-child interactions as such. Children’s cognitive performance was assessed by three subscales of the Wechsler Preschool and Primary Scale of Intelligence (WPPSI). The results of the analysis of covariance (ANCOVA) indicated a significant difference between the experimental and control group. The findings support the effectiveness of the parent training program for enhancing cognitive performance in preschoolers.

Understandings of child development indicate that child’s learning capacities along with mental health bases are established in childhood. In this sense, any impairment during the processes of childhood development can negatively affect children’s capacities to learn and communicate with others (Zigler, Shonkoff, & Meisels, 2000). Moreover, the all-round and optimal child development is the product of the interaction of multiple factors and the dynamic exchanges of biological structures, genetics, and environmental features. One of the important factors in this case is a child’s relationship with 'others' (especially with their mother) (Mäntymaa, 2006). The quantity and quality of mother-child interactions can influence many features of child development, including social, emotional and cognitive ones. However, while nowadays the world is rapidly changing and children need to acquire knowledge and many skills to be qualified individuals, many parents are concerned about their children's cognitive development and want to know whether and how they can improve their cognitive abilities.

A large number of theories and studies over the last three decades have highlighted the role of parents in promoting cognitive development in children. In particular, researchers suggest that if human interaction with others follows certain features, it can contribute the most to the cognitive development of children. Researchers have put forth effective strategies and parental effective behaviors leading to improvements in thinking skills and cognitive processes in children. They include *scaffolding* (see Wood, Bruner, & Ross, 1976), *responsiveness* (see Bornstein & Tamis-LeMonda, 1990) and *mediated learning* (see Feuerstein, Feuerstein, Falik, & Rand, 2002; Klein, 1996; Tzuriel, 1996, 2001, 2011). Although numerous studies have validated the significance of parent-child interactions in a child's cognitive performance, little research has already been conducted to see whether training parents to improve the quality and quantity of parent-child interactions can contribute to children's cognitive abilities. To answer this question, a group parent training program was developed in this study based on the theory of the *Structural Cognitive Modifiability (SCM)* and the *Developmental, Individual Differences, Relationship-based model (DIR)*.

## Theoretical Framework

### Theory of Structural Cognitive Modifiability and Mediated Learning Experiences

The theory of structural cognitive modifiability (SCM) (Feuerstein, Feuerstein, & Falik, 2015) is one of the groundbreaking theories of this training program. The major premise of this theory is to introduce intelligence not as a fixed structure, but as a variable capable of change and improvement. Feuerstein considers man as a system open to change in which the cognitive structures are allowed for change and modification (Feuerstein, Feuerstein, & Falik, 2015). He defines SCM as the human capacity to learn from new experiences, learning opportunities, and ability to change the cognitive structures, which seeks to accommodate the ever-changing needs of life (Mentis, Dunn-Bernstein, & Mentis, 2008). Feuerstein assumes that there are only a few people who can realize their full potential in individual learning contexts; however, using mediated learning experiences can help people not only reach their learning potential but also modify the actual cognitive structures (Feuerstein, Falik, & Feuerstein, 2013). In mediated learning experience (MLE), learning takes place through a more experienced individual (usually a parent) who mediates between the child and the world of stimulants. A mediator is a person who learns about the needs, interests, and capacities of the child and plays an active role in creating the child’s environment; in fact, a mediator changes stimulants in various ways, adjusts their frequency, order and intensity, and consciously raises children’s interest and curiosity to foster their basic cognitive functions (Feuerstein & Falik, 2010).

Now the question that comes to mind is what features an interaction should include to be a mediated learning experience. Lidz (2002) has defined twelve features for mediated behavior in the adult-child interaction which leads to the promotion of higher cognitive functions in children. The first five MLE criteria are operationalized and observed in interactions of mother-child (Klein, 1996; Klein & Alony, 1993; Tzuriel, 1999, 2001, 2011), peer-assisted learning (Tzuriel & Shamir, 2007, 2010), siblings (Klein, Feldman, & Zarur, 2002) and teacher-student instruction (Remer & Tzuriel, 2011). The first five MLE criteria operationalized for research are as follows: 1) mediation of intentionality, including the mediator’s deliberate attempt to teach children through involving them in various assignments and changing the environment and stimulants so that he/she can capture children’s attention; 2) mediation of meaning, including the mediator’s use of gestures, sounds, facial expressions and body movements to enliven the interaction, and highlight what needs to be noticed; 3) mediation of transcendence, that is, to bridge across the ideas, experiences and events with the goal of expanding learning environment and creating new learning; 4) mediation of task regulation, that is, the mediator helps children consider different solutions, evaluate them and adopt the best one, and if necessary, teaches them the basics of planning and problem-solving; 5) mediation of praise and encouragement, including the mediator’s attempts to provide children with opportunities for success and praise, and encourage them at the time of success and also give them feedback as to which characteristics of the child’s approach in carrying out the task deserves encouragement.

According to the theory of SCM, parents have become aware of the possibility of enhancing learning potential in children based on the advantage of cerebral plasticity (Feuerstein, Falik, & Feuerstein, 2013) and the prominent role they play in helping children learn. They understand that they can assist in making children’s learning potential flourish. By enriching their interactions with children using the features of mediated interactions (i.e. mediation of intentionality, mediation of meaning, mediation of transcendence, mediation of task regulation, and mediation of praise and encouragement), they also learn how to improve their children's cognitive functions. However, for parents to be able to function as effective mediators for children, they are required to have a comprehensive understanding of their children's needs, interests, capacities and cognitive strengths and challenges. As such, the DIR model can help them achieve such an understanding.

### Developmental, Individual Differences, Relationship-Based Model (DIR)

Greenspan and Wieder (2006) have illustrated the course of emotional-functional development from birth to adulthood after several years of careful observation of early emotional signs, cognitive and motor processing differences, the relationship of caregiver with child, and family interaction patterns in children (both children with normal and impaired development). As a result, they have introduced the Developmental, Individual Differences, Relationship-based (DIR) model with a bio-psycho-social approach to human development. The model suggests that human development is driven by the interaction of three dynamic components: 1) children’s biological and genetic conditions, including their abilities or challenges in sensory and motor processing; 2) children’s social environment, including their family and cultural characteristics and other environmental factors; and 3) patterns of children’s interactions with 'others' influenced by the dynamic interaction of the two aforementioned components and determined by children's developmental outcomes in all emotional, social and cognitive aspects (Greenspan & Wieder, 2006). The principle assumption forming DIR or human integrated development model (see Aminyazdi, 2012) is the vital impact that a child’s emotional relationship with the caregiver and their emotional and social interactions with 'others' can have on their psychological development (Greenspan, 2007).

DIR model has numerous implications for enriching child-parent relationships, which aims at improving the child’s learning. As an example, this model assumes that each child influenced by genes and factors both during pregnancy and after birth possesses a unique biological and psychological structure. These individual differences in sensory modulation, sensory processing and motor planning lead to differences in child’s cognitive abilities and a variety of performance in learning situations. Parents’ awareness of the unique biological characteristics of their children helps them adjust their interactions with the children’s individual differences, which provides them with learning experiences of higher quality as such (Aminyazdi, 2012). In addition, the DIR’s great emphasis on the importance of emotions suggests that parents’ attention to the emotional content of their relationships with children fine-tune the child-parent interactions more effectively. For example, if the parent is warm, calm and responsive, the child will be more attracted to the child-parent interactions, as parents need to consider children’s emotions for effective mediation. In fact, to teach their children, they should choose stimulants which arouse more interest, for instance.

## The Parent Training Program

Drawing upon the two theoretical approaches described above, it is inferred that the parent-child interactions play a key role in improving children’s cognitive development and learning. In fact, an optimal parent-child interaction can be described by combining the DIR model with the theory of SCM. Such an interaction can significantly contribute to the improvement of a child's cognitive development and learning. That is, in addition to being warm, lovely, attentive, and not restrictive, the parent functions as a mediator between the child and the environment and consciously provides the experiences based on the child’s individual characteristics, level of development, interests and emotions, which results in better learning.

In the present study, a group-parenting program was developed by the present researchers for mothers of normal preschool children in order to raise the quantity and quality of the optimal mother-child interactions. Parent training programs are expected to not only enhance the parent’s knowledge and parenting skills but also to assist them in mastering the obtained skills. In addition to raising the mothers’ awareness of children’s fundamental issues along with the change and modification of their attitudes towards children’s learning, mothers learn strategies to boost the quantity and quality of their interactions with their children in this program with the aim of improving their children’s cognitive performance.

Changing mothers' attitudes and their implicit theories about intelligence and learning abilities is one of the main objectives of the program. Dweck (2012) calls the individuals' implicit theories *mindsets*. Mindsets are people’s lay beliefs about the nature of human attributes, such as intelligence (Yeager & Dweck, 2012). Some people hold a fixed mindset (or an entity theory) and believe that human attributes are simply fixed traits. For example, they might believe that each person has a fixed amount of intelligence and cannot change it. In contrast, other people hold a growth mindset (or an incremental theory). For example, they may believe that all people, no matter who they are, can become substantially more intelligent through their effort and education (Dweck, 2017). Believing a growth mindset can enhance mothers' motivation to help their children boost their cognitive abilities.

The treatment objectives are outlined in Table 1.

**Table 1:** Sessions' Objectives

Session	Objectives
1.	To become familiar with members and their goals and expectations. To learn about the role of parent in child development. To learn about the efficacy and necessity of parent training program.
2.	To learn about the possibility of promoting children’s cognitive performance through mediational learning experiences.
3.	To acquire knowledge about children’s biological and genetic characteristics, which lead to individual differences in learning.
4.	To practice how to regulate environment, behaviors, and child-raising practices on the basis of children’s individual characteristics.
5.	To familiarize with children’s interests, needs, cognitive strengths and challenges, and emotional tendencies through play. To learn about Floortime play.
6.	To practice Floortime play techniques.
7.	To learn about mediational strategies aimed at enriching daily parent-child interactions.
8.	To learn how to transform daily situations into enjoyable activities and opportunities for teaching, solving multiple cognitive problems, and strengthening children’s language, memory, knowledge, and basic operations required for logical thinking via mediational learning strategies.
9., 10., 11.	To practice the obtained skills.
12.	To summarize and overview all the sessions.

## Method

### Design

This study was a quasi-experimental design which compared pretest and post-test measures. Participants were divided into an experimental group and a control group.

### Participants

The parenting program designed in this study aims for both mothers and fathers. However, only mothers were involved and contributed in this study due to some administrative constraints and the fact that mothers in Iranian culture are mostly responsible for child care and education at this specific age range. Mothers living in Mashhad, Iran with a preschool-aged child and meeting the inclusion criteria for the study were invited to participate in it. The criteria for their selection include the following: (1) mothers should have children aged 4-6years old; (2) mother should have nine years education or more.

To assure the homogeneity of the sample, single-parent mothers and also mothers and children with identified cognitive and learning problems were excluded from the early sample. A convenience sampling of 29 couples of mothers and their children were recruited during the school summer holidays in 2015. The mothers consciously (knowing the objectives of the study and how to do it) and voluntarily completed a form to show their willingness to participate in the project. As such, sixteen mothers were selected together with their children for the experimental group while thirteen couples of mothers and children were selected as the control group. The demographic characteristics of the participants are shown in Table 2. There were no significant differences between the experimental and control groups in terms of demographic characteristics.

### Measure: The Wechsler Preschool and Primary Scale of Intelligence (WPPSI-R)

Wechsler Intelligence Scale is one of the most frequently used instruments for measuring a child’s cognitive ability. In this study, in order to measure the child’s cognitive performance, a combination of the three subtests of block design, arithmetic and similarities of the Wechsler Preschool and Primary Scale of Intelligence (WPPSI) was utilized. The scores from the subscales of this test can be added up and the final score can be placed on an interval scale. The validation and Standardization of the test in Iran has been conducted by Razavieh and Shahim (2000).

### Procedure

A pre-test was administered right after selecting and placing the participants of the control and experimental groups. Mothers in the experimental group participated in the training program, including 12 group-sessions. For nine sessions the mothers participated alone, whereas for three exercise sessions mothers brought their children for further practice of the acquired skills every session.

The control group received no treatment while the experimental group was attending the sessions. A post-test was then administered two weeks after the last training session.

**Table 2:** Demographic Characteristics of Participants

	Experimental group (*n* = 16)	Control group (*n* = 13)
	Number (%)	Number (%)
Gender of the child- boy	10 (62.5)	9 (69.2)
Gender of the child- girl	6 (37.5)	4 (30.8)
Mother’s education- 12 years or less	11 (68.7)	8 (61.5)
Mother’s education- 13 years or more	5 (31.3)	5 (38.5)
father’s education- 12 years or less	10 (62.5)	10 (76.9)
father’s education- 13 years or more	6 (37.5)	3 (23.1)
Mother in employment	4 (25.0)	3 (23.1)
Mother not in employment	12 (75.0)	10 (76.9)
Father in employment	15 (93.7)	12 (92.3)
Father not in employment	1 (6.3)	1 (7.7)
	*M* (*SD*)	*M* (*SD*)
Age of the child	5.20 (0.75)	5.28 (0.74)
Age of the mother	28.51 (3.56)	29.65 (3.21)
Age of the father	33.81 (3.49)	34.23 (4.32)

### The Intervention

The experimental group attended a parent-training program designed by the authors, consisting of twelve weekly 2-hour group sessions. The methods applied in the sessions included instruction by a trainer, sharing experiences, group discussions and role-plays. Each session (except session 1) began with a review of the previous session and a discussion of the assigned homework. This was followed by mini-lectures on the new topics to be taught by the trainer along with group discussions and role-plays. Finally, after summarizing the exchanged ideas, homework was assigned to master the skills and acquire further knowledge. The session ended when the instructor provided a short account of the shared viewpoints. There were 12 two-hour sessions altogether; the objectives of sessions are listed in Table1 and content of the sessions are given in the following:

*Session 1:* At the beginning of the session, members introduced themselves and the instructor explained the rules of group discussions. Next, the instructor elaborated on the importance of the role of mothers in child development, and the efficacy and necessity of participation in a parent training program. The session closed with a brief explanation of the objectives, method and plan of future sessions given by the instructor.

*Session 2:* In accordance with the principles of the theory of SCM and mediated learning experiences, this session opened with raising mothers’ awareness of the possibility of enhancing children’s learning potential (cerebral plasticity) and their leading role in helping children learn. The instructor then explained that mothers need to have a thorough understanding of their children's needs, interests, capacities and cognitive strengths, and challenges in order to be efficient mediators.

*Sessions 3 and 4:* At first, the unique biological and genetic characteristics of the children determining their individual differences in learning situations were explained. Mothers were then assisted to acquire an image of their children’s individual characteristics. In the end, mothers were informed that they should regulate and tailor their environment, behavior, and child-raising practices to their children’s individual characteristics to be considered as an efficient mediator for children. This is, however, made possible by learning about each child’s individual characteristics.

*Sessions 5 and 6:* The sessions opened with the trainer, defining ‘play’ as an enjoyable spontaneous activity for children and listing the functions of the ‘play’ in improving their development and learning. Next, the parent-child ‘Floortime play’ was introduced. ‘Floortime’ is a quality of interactions abundant with plays in which parents strike up warm relationships with their children based on their individual characteristics and interests, follow their leadership (emotional interests), help them learn communicate with others, and finally challenge them to expand their emotional, social and cognitive capabilities (Pajareya & Nopmaneejumruslers, 2011). In the end, the mothers practiced their skills in ‘Floortime’ through role-plays and make use of the trainer and other mothers’ feedback.

*Sessions 7 and 8:* At that stage, having acquired the knowledge about their children’s individual characteristics, interests, and emotional tendencies, mothers could help their children with their learning by applying the mediated learning skills in order to improve and enrich their interactions. In session seven and eight, mothers became familiar with the features of the mediated learning interactions. As such, the mediator transformed everyday situations (such as easting, shopping, etc.) into enjoyable activities opportunities for teaching, solving multiple cognitive problems, and strengthening children’s language, memory, knowledge, and basic operations required for logical thinking (categorization, classification, spatial visualization, etc.).

*Sessions 9, 10, and 11:* At the beginning of those sessions, the trainer gave some examples on how to utilize the mediated learning skills to transform ordinary real-life situations (such as cooking) into a mediated learning experience. The mothers then raised their questions and practice the skills through group discussions and role-plays.

*Session 12:* In the last session, after a general overview and summary of all the sessions, the mothers raised their questions, views and experiences, and receive feedback from the instructor and other mothers.

This intervention program was conducted by an educational psychologist (the third author) under the supervision of the first author who is a developmental psychologist. The control group was not offered any extra services while the experiment group attended the parent-training program. The control group was offered a book (developed by the first author) after the experimental group had completed the program. It should be noted that the tester who administered the pre-test and post-test was blind to the treatment assignment.

### Data Analysis

Descriptive statistics, including mean and standard deviation, were used to describe the results of the pre-test and post-test. To compare the post-treatment results of the experimental and control groups, the analysis of covariance (ANCOVA) was used. Prior to the use of the analysis of covariance, it was assured that all its assumptions were met. The Statistical Package for Social Sciences (SPSS) software, version 22.0 for Windows, was used for data analysis.

## Results

Table 3 depicts the descriptive results derived from the pre-test and post-test of the children’s cognitive performance variable. As shown in the table, the mean score of the cognitive performance variable has increased 2.07 for the control group and 7.62 for the experimental group.

**Table 3:** Mean and Standard Deviation of Pre-Intervention and Post-Intervention Tests

	Pre- intervention		Post- intervention		Pre and Post intervention Difference (D)
	*M*	*SD*	*M*	*SD*	
Experimental group (*n* = 16)	33.48	6.46	41.06	6.05	7.62
Control group (*n* = 13)	32.85	11.14	34.92	10.24	2.07

To study the statistical significance of the difference between the means, the analysis of covariance (ANCOVA) was conducted. Prior to use of ANCOVA, it was assured that assumption of normality using Shapiro-Wilk’ W test [pretest: *p* = .156, posttest: *p* = .776], assumption of homogeneity of variances using Leven’s test [pretest: *F*(1, 27) = 3.114, *p* = .089 posttest: *F*(1, 27) = 2.127, *p* = .156], and assumption of homogeneity of regression slopes, *F*(1, 26) = 1.651, *p* = .210, were met. There was a significant difference between the two groups regarding the children’s cognitive performance, *F*(1, 26) = 17.48, *p* < .001, and the effect size (the partial Eta Squared value) compared with Cohen’s guidelines is moderate (0.452).

## Discussion

There is a general consensus from cognitive developmental research that children benefit from new learning experiences through the advantage of cerebral plasticity (Bonnier, 2008). In addition, a large body of theories and practices support the idea that adult-child interactions are of central importance in this process (Farah et al., 2008; Rao et al., 2010; Vygotsky, 1980; Wood, Bruner, & Ross, 1976). A large number of studies have shown that the quality of mother-child interactions is a direct predictor of children’s performance in cognitive tests and problem-solving situations (see Tzuriel, 2013).

Theory of structural cognitive modifiability considers cognitive abilities, including thinking and problem-solving skills, not fixed structures but variables which can be modified and improved if children have access to an efficient mediator. Kozulin et al. (2010) and Salas et al. (2010) have demonstrated that providing children with mediated learning experiences by teachers improves their cognitive abilities. In another study, a year-long caregiver training program based on SCM theory, mediational intervention for sensitizing caregivers (MISC), was effective in teaching Ugandan caregivers to enhance their children's cognitive development through practical and sustainable techniques applied during daily interactions at home (Boivin et al., 2013).

The hypothesis of the current study tested the impact of enriching mother-child interactions via a parent training program (as explained earlier in the paper) on the improvement of children' cognitive performance. The analysis of the research data revealed that the parent-training program significantly improved children's cognitive performance. To elaborate further on the result, the following can be inferred:

Based on mindset theory (Dweck, 2012), as mothers became aware of the advantage of neuroplasticity and the prominent role of parent-child interactions in children’s cognitive development and their implicit theories of intelligence change to a growth mindset, they became motivated to provide new learning experiences for their children.

Klein and Rye (2004) state that teaching mediational strategies to parent increases their intentional teaching behaviors. In the same vein, Klein and Alony (1993) have shown that even three years after receiving the mediation training the mothers in the experimental group have significantly more intentional teaching behaviors in comparison with those in the control group. In this study, mothers in the experimental group practiced to use different techniques to capture and maintain children’s attention to colors, shapes, animals, numbers, similarities, differences, etc. during their daily interactions. Apart from expanding children’s learning opportunities in quantity, the quality of mother-child interactions improved as mothers learned how to match the interactions with their children's individual characteristics and to consider their emotions and interests as such.

With an increase in the quantity and quality of the mediated mother-child interactions, the children’s knowledge increases; they learn the fundamental logical thinking skills (i.e. categorization, object assembly, comparison, etc.) and improve their language abilities (i.e. range of vocabulary, language comprehension, etc.) as such (Feuerstein & Falik, 2010). Additionally, appropriate parent-child interactions raise children’s curiosity, create new questions for them, in which case they make children pay closer attention to their environment, experiment, question, compare, and consider similarities and differences, and make connections between environmental stimulants (Klein, 2000). Thus, children can also learn from their direct experiences with the environment and use their skills in new situations. Furthermore, since these interactions are in line with children’s interests, needs, cognitive strengths and challenges, and the individual characteristics mainly carried out through play-like activities, they increase children’s motivation to learn (Pintrich, 2002).

Slavin (2006) claims that the best way to improve children’s performance in cognitive problem-solving is the children’s extensive practice in different situations along with receiving feedback from adults. With an increase in the mother-child mediated interactions, children are provided with various situations where they can solve different cognitive problems and benefit from their mother’s feedback. In addition, children can observe their mother’ reaction to the problems and the ways they solve the problems, and then they internalize them as such.

Another possible explanation is that learning through real-life experiences provides strong possibility of transferring learning to other situations (Santrock, 2004), whereas enriched parent-child interactions give children the opportunity for learning through real-life situations, such as cooking, bathing, shopping, etc.

Finally, consistent with the findings of studies showing the role of mediated learning experiences in enhancing children’s meta-cognition (Adi‐Japha & Klein, 2009; Feuerstein & Falik, 2010), the enriched interactions lead to improved meta-cognitive skills in children. This is because parents, as the subject of the parent-training program, learn and practice to teach their children how to plan for what they want to do, ask for help if necessary, talk to themselves while problem-solving, encourage themselves, and continue their effort to the end of a task.

The overall results offer evidence that the parent-training program was effective in enhancing children’s cognitive performance.

Although the parent-training program was first developed for Iranian families in Mashhad, it might potentially be useful for other Iranian communities. It could also be used as a basis for similar programs in non-Iranian communities. However, it is worth noting that although the results of the present study were promising, more research would be needed to establish the evidence-basis of the Parent-training program used in the study.

### Limitations

This study is limited in terms of generalizability due to the small convenience sample used. Another limitation of the study is that it was not possible for fathers to participate in this study due to some administrative constraints. It is suggested that the possibility of the pursuit of the project over a longer period of time along with the investigation of the effectiveness of teaching parents be considered in future studies. Another limitation of this study is that a static tool was adopted to assess children's cognitive performance while dynamic tools are more compatible with cognitive variables targeted in parent training-programs. It is recommended that researchers consider the possibility of gaining access to a dynamic tool to obtain more reliable data for future studies.

### Implications for Research and Practice

In the present study, the program was offered to parents of children without any cognitive or learning problems; however, the effectiveness of the program for children with different types of learning problems would need to be examined. The effectiveness of the program for children less than four years old should also be investigated. The most important implications for practice from this study relate to the determination of the effectiveness of parental training program to enhance children’s cognitive performance. On the other hand, as the results supported the leading role of mother-child interactions in children's cognitive performance, the enrichment of mother-child interactions through parent training programs is a matter of utmost importance.

### Conclusion

This study addressed a gap in the literature by providing empirical evidence that the group-parent training program is effective in promoting children’s cognitive performance. Most importantly, this study highlights the significance of parent-child interactions in preschool children’s cognitive performance.
